# Atomic force microscopy study of DNA flexibility on short length scales: smooth bending versus kinking

**DOI:** 10.1093/nar/gku1192

**Published:** 2014-11-20

**Authors:** Alexey K. Mazur, Mounir Maaloum

**Affiliations:** 1 UPR9080 CNRS, Université Paris Diderot, Sorbonne Paris Cité, Institut de Biologie Physico-Chimique, 13, rue Pierre et Marie Curie, Paris 75005, France; 2 Institut Charles Sadron, CNRS–University of Strasbourg, 23 rue du Loess, BP 84087, 67034 Strasbourg Cedex 2, France

## Abstract

The apparently anomalous flexibility of DNA on short length scales has attracted a lot of attention in recent years. We use atomic force microscopy (AFM) in solution to directly study the DNA bending statistics for small lengths down to one helical turn. The accuracy of experimental estimates could be improved due to a large data volume and a refined algorithm for image processing and measuring bend angles. It is found that, at length scales beyond two helical turns (7 nm), DNA is well described by the harmonic worm-like chain (WLC) model with the bending persistence length of 56 nm. Below this threshold, the AFM data are also described by the WLC model assuming that the accuracy of measured bend angles is limited by the physical width of the double helix. We conclude that the double helical DNA behaves as a uniform elastic rod even at very short length scales. Strong bends due to kinks, melting bubbles and other deviations from the WLC model are statistically negligible.

## INTRODUCTION

The ability of duplex DNA to loop, fold and wrap around proteins plays important roles in many biological processes. Different facets of this complex property are intensively studied since 1970, but it still remains puzzling. Many good reviews of DNA bending *in vivo* and *in vitro* have been published in different years (1–4). The problem that has attracted much attention in recent years concerns the structure of strongly bent DNA. This issue was raised long ago. Crick and Klug suggested that strong bends, necessary for DNA compaction in the limited space of cells and viruses, occur when base pair stacking is disrupted, probably at weakest steps, which gives free hinges or ‘kinks’ (5). Somewhat later, it was shown by calculations that strong bends in duplex DNA can be achieved without destacking, by subtle low-energy fluctuations of bond angles and torsions (6). This second picture corresponds to the elastic rod or worm-like chain (WLC) model earlier proposed in polymer physics (7–9). The WLC model proved to be most adequate description of experimental data on long DNA. However, the long-chain predictions of the WLC model are essentially independent of the details of local bending. For lengths shorter than the bending persistence length (*l*_*b*_ = 50 nm), these details should play a larger role and the agreement with experiment can well disappear (10). In fact, for short and intermediate length scales, deviations from predictions of the WLC model were uncovered using different experimental methods (11–16). According to these data, short DNA looks much more flexible than the WLC prediction, which was explained by assuming inherently high ‘kinkability’ of the double helix (17–24). However, these conclusions are questioned and vigorously debated in the literature (25–35). An insightful review devoted to this problem was published recently (36).

Atomic force microscopy (AFM) is a powerful single-molecule technique that can directly observe DNA when adsorbed onto supporting surfaces. This method has been used many times for probing DNA bending in different conditions (37–44), and potentially it can check the correspondence between the WLC model and DNA bending at all length scales. Earlier studies showed that, in appropriate conditions, the DNA molecules can be equilibrated on the surface so that statistics of bend angles agree with the WLC predictions with very good accuracy in spite of relatively strong surface–DNA interactions (45,46). With reduced DNA length, the experimental errors involved in measuring bend angles grow and special care is required for evaluating bend angle distributions in short DNA. The first AFM investigation of DNA flexibility on short length scales was reported by Wiggins *et al.* (13). It was found that for chain lengths beyond 30 nm the equilibrium statistics of bend angles and end-to-end distances agree with those of the WLC model, but for shorter lengths the populations of strongly bent conformations are much higher than the WLC predictions. Based upon the shapes of the experimental probability distributions it was concluded that the DNA double helix is intrinsically kinkable, that is, it is kinked rather than bent smoothly even for small angles. Quantitatively, these results were accounted for by the linear sub-elastic chain (LSEC) model (13). The conclusions of this first report were entirely confirmed in another AFM study where bending was measured in very high resolution images of DNA minicircles (15).

In our recent paper, the statistics of DNA bending at short length scales were evaluated by using AFM in solution (47). We confirmed the apparent short length kinkability corresponding to the LSEC model, but found some evidences that this effect could be an artifact of the analysis of AFM images. By changing the image processing parameters we found that bending fluctuations in DNA absorbed on a plane in solution are Gaussian and well described by the WLC model at all length scales beyond three helical turns (10.5 nm). Below this threshold strong apparent deviations from the WLC behavior were still observed, but the origin of these features could not be clarified because the algorithm used for tracing DNA contours evidently required improvements.

In the present study, we use the AFM method to get an insight into the origin of the apparently non-Gaussian behavior of short DNA and to estimate the relative contribution of kinking. To this end, we improved the semi-automatic tracing method used for processing AFM images and added new data obtained in different conditions. We show that the accuracy of the previous measurements was indeed limited by the tracing procedure. With the refined algorithm the short length limit of reliable agreement of AFM data with the WLC model is reduced from 10.5 to 7 nm (two helical turns). Below this threshold, the AFM data are also described by the WLC model if the accuracy limit due to the finite width of the double helix is properly taken into account. We conclude that, in these conditions, the double helical DNA behaves as a uniform elastic rod even at very short length scales. Strong bends due to kinks, melting bubbles and other deviations from the WLC model are statistically negligible.

## MATERIALS AND METHODS

### AFM experiments

We used the AFM method to evaluate DNA bending at short length scales. Linear DNA with a fixed length of 4363 bp was obtained by cutting supercoiled PBR322 plasmid with EcoRI restrictase. Most experiments were performed in a solution containing 10-mM tris-HCl buffer, pH 7.5, supplemented with 1-mM MgCl_2_, to a final DNA concentration of 1 mg/ml. 200 ml of this DNA solution was injected in AFM liquid cell and DNA molecules adsorbed onto freshly cleaved muscovite mica at room temperature. Images were collected using a Nanoscope 8 (Bruker) operated in tapping mode in solution, with a pixel size (grid spacing) of 1.95 and 3.9 nm, with the standard line scan rate of 1.4 and 0.7 Hz, respectively. Ultrasharp non-contact silicon cantilevers Multi75Al (NanoAndMore) were driven at oscillation frequencies in the range of 20–26 kHz. During AFM imaging, the force was reduced in order to avoid dragging of DNA by the tip. Integral gain was adjusted to give sharp images. Images were taken without on-line filtering and were subsequently processed only by flattening to remove the background slope.

The AFM images of DNA were transformed into discrete chains under visual control as follows. A custom implementation of the algorithm by Wiggins *et al.* (13) was used for initial tracing with link length *l*_0_ equal to 7 or 14 nm. The subsequent refinement was made as described further below using the same or smaller *l*_0_ values. The DNA bending statistics were evaluated for two independent sets of AFM images. In the first one, analyzed in the previous report (47), the total contour length of DNA measured with *l*_0_ = 7 nm was ∼340 μm (∼1016 kb). The second set, scanned with two times lower resolution, involved ∼305 μm of DNA (∼910 kb). For statistical estimates the contours were divided into fragments so that every measured angle was counted only once.

### Tracing algorithm

Not all of the many tracing algorithms proposed earlier (45,46,48,49) are applicable for statistical sampling of bend angles in short DNA. The method used in the previous such studies was developed by Wiggins *et al.* (13). This is an iterative prediction–correction schema. For initialization, two points are placed manually at one of the chain ends. The prediction is made by stepping forward in the direction of the previous link. The subsequent correction in the perpendicular direction is made using the starting point as the origin of coordinates and computing a new direction as

\begin{equation*} \vec{X}\equiv \int _0^{10 {\rm nm}}{\it ds}Z(\vec{x})\vec{x}(s), \end{equation*}

where $\vec{x}$ is the position vector in plane, $Z(\vec{x})$ is the local height and the integral is taken over the perpendicular segment. The iteration is repeated three times using vector $\vec{X}$ as the new prediction direction. After that a final step forward is made with the required link length. The image height $Z(\vec{x})$ is computed at any point by interpolation from the pixel grid, that is, the algorithm operates at a sub-pixel resolution.

The above algorithm is simple, rapid and suitable for processing a large number of DNA images necessary for good statistics. However, it uses a manually set starting point and search direction, therefore, its result is a bundle of contours rather than a single line. It appeared that the spread of this bundle dramatically grows when the tracing link length is reduced (47). In bad cases the bundle width reaches a few pixels, that is, the divergence exceeds the instrument resolution. To correct this problem the above algorithm was supplemented with a refinement step in the spirit of some earlier methods (45,48). To this end, on every link of already computed trace, a set of points is considered separated by half-pixel intervals. At each such point a perpendicular segment is constructed and the maximum of $Z(\vec{x})$ is found. This gives a large set of points approximately at the centerline of the DNA trace. A broken line through these points may involve acute angles because in strongly bent regions searches in the perpendicular direction may find maxima ahead or behind the reference link. Therefore this broken line is smoothed by iteratively removing points that produce acute angles. After that the refined trace is computed by stepping along the broken line with the necessary link length. These computations are rapid and they can be repeated several times or until convergence according to some criterion.

**Figure 1.**
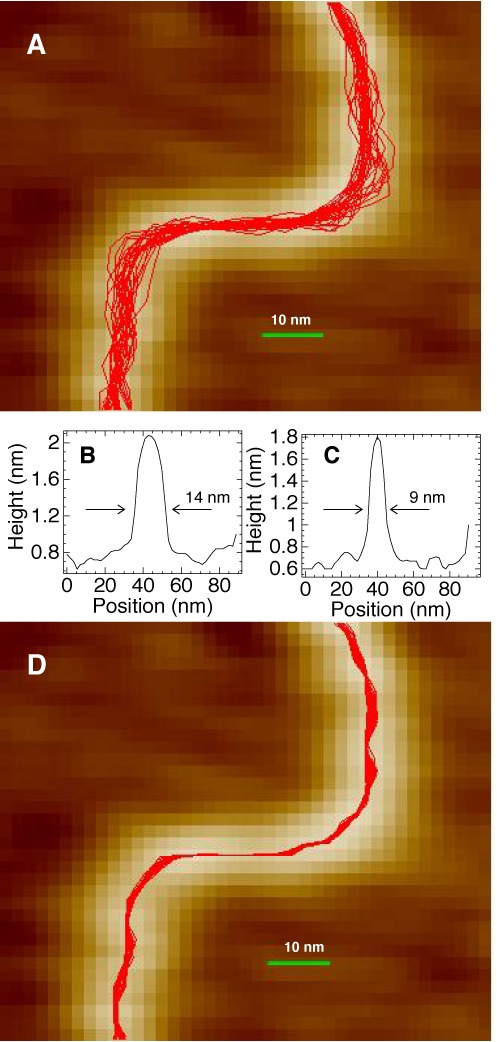
(**A**) A zoomed out view of a DNA trace on AFM scan with a bundle of contours computed with the link length *l*_0_ = 3.5 nm. (**B, C**) Two cross-sectional profiles measured for the DNA molecule from panel (A). Trace B corresponds to the zone where the bundle width is maximal. Trace C was taken elsewhere and it is close to average. (**D**) The same image as in panel (A) after the refinement procedure described in the text.

A representative example of the effect of the refinement step is shown in Figure 1. Panel (A) shows an AFM image of a DNA fragment with 30 traces computed by the original algorithm using opposite directions and different manually set starting points. In this region the DNA molecule is strongly bent and its AFM image is wider than average, which explains a relatively strong divergence of the computed traces. Panels (B) and (C) demonstrate, however, that the cross-sectional profiles usually have well-defined maxima regardless of the local image width. Therefore, the spread of the computed contours could be reduced if the height maxima were better localized. Panel (D) displays the contours form Panel (A) after the refinement step. The width of the bundle still is not constant because the cross-sectional profiles fluctuate as exemplified by Panels (B) and (C). However, the width is significantly reduced and now it rarely exceeds one pixel. Moreover, in many regions it is less than one pixel because the tracing algorithm operates at sub-pixel resolution by using interpolation from the pixel grid.

### Monte-Carlo simulations of AFM data

The AFM data were compared with Monte-Carlo (MC) simulations of a planar discrete WLC model. A phantom chain was considered without the excluded volume effect. The bend angles were sampled directly from appropriate Boltzmann distributions. To get a feel of statistical errors the DNA length and the volume of sampling were similar to those in experiment. The link length *l*_0_ in the digitized AFM DNA contours is always larger than 1 bp. For short chains this gives a significant bias with respect to the underlying DNA (47). To take this into account, MC simulations of the WLC model were performed with one bead per bp, but the resulting chain configurations were resampled by stepping along MC bead positions with fixed strides corresponding to link lengths in AFM data. These new contours were processed in the same way as experimental data to generate reference WLC distributions.

## RESULTS AND DISCUSSION

### Kinking versus smooth bending

The interplay between kinking and smooth bending is quite simple if we consider an elastic rod confined in a plane. Suppose we study the ensemble of conformations with a certain bend angle. The rod can be considered as a concatenation of flexible segments, therefore, the bend angle and the energy of bending can be computed as a sum over internal segments. In thermal equilibrium, all thermodynamics observables are determined by conformers in the vicinity of the global energy minimum (8). Suppose the bending energy of a segment is described by function *E*(*θ*). If it is harmonic the global minimum is reached when the curvature is evenly distributed along the rod. This follows from the simple fact that, for any positive *a* and *b*, (*a* + *b*)^2^ > *a*^2^ + *b*^2^. This conclusion can be expanded to arbitrary convex functions because any convex curve can be divided into small intervals and approximated by parabolas. The same reasoning leads to a conclusion that when *E*(*θ*) is concave the bending should be concentrated in a single point (kinked rod).

**Figure 2.**
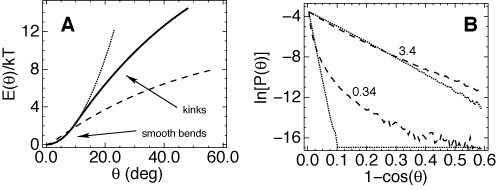
(**A**) Three alternative profiles of the bending energy for a discrete WLC model with the macroscopic bending flexibility corresponding to experiment (*l*_*b*_ = 50 nm). (**B**) Equilibrium probability distributions of DNA bending computed by BD simulations with two potentials shown in panel (A). The distributions are shown for DNA lengths 0.34 and 3.4 nm (shown near the plots). The dashed and dotted lines correspond to those in panel (A).

A single base-pair step (0.34 nm) is the minimal fragment of duplex DNA for which the bend angle can be measured and the bending energy described by a potential *E*(*θ*). Figure 2A displays three possible profiles of *E*(*θ*) for a discrete WLC model with one bead per base pair and the macroscopic bending persistence length of 50 nm. The harmonic energy (the dotted line) grows very rapidly, which is impossible beyond a few kT because the estimated lower limit for melting and kinking is around 10 kT (36). The harmonic approximation probably fails well below this limit because the straight minimum energy conformation is maintained by non-covalent interactions that break down with small changes of atom–atom distances. A more probable profile is shown by the solid line. It is convex for small *θ* and becomes concave beyond a certain angle *θ*_0_ arbitrary chosen as 10° in Figure 2A. The solid and dotted curves diverge only for large energies; therefore, this difference does not affect the measured *l*_*b*_ values. The flex point at *θ*_0_ can be due to loosened hydrogen bonding or stacking, but not necessarily. This is a generic energy profile of a structured object, and the foregoing reasoning explains why such objects are smoothly deformed by small forces and break down when the external load exceeds a threshold. In the DNA case, destacking or local melting would give another energy minimum with *θ* > *θ*_0_, but it is not strictly required. If the profile of *E*(*θ*) has a flex point at *θ*_0_, larger bends should be predominantly kinks. In small DNA loops they appear when the average curvature corresponds to *θ* ≈ 6° or larger (50).

The dashed profile in Figure 2A demonstrates the case of extreme kinkability. This potential is defined as

(1) \begin{equation*} E(\theta ) = \left\lbrace \begin{array}{cr}q\theta ^2 & \theta <\theta _0\\ q\theta _0^2-\frac{q}{k}\theta _0(\theta _0-\pi )\left[1- \left(\frac{\theta -\pi }{\theta _0-\pi }\right)^{2k}\right] & \theta \ge \theta _0 \end{array} \right. \end{equation*}

with *θ*_0_ = 2°, *k* = 2 and *q* = 110 kcal/mole. It is convex around zero to avoid singularity, but remains concave beyond a negligible *θ*_0_ without additional energy minima. With this profile, even small bends represent predominantly kinks rather than smooth deflections. In the previous report (47) we showed that it corresponds to the LSEC model (13). Figure 2B displays the bend angle distributions for harmonic WLC (dotted lines) and LSEC (dashed lines) models computed by Brownian Dynamics (BD) simulations (47,51). The distributions corresponding to the solid line in Figure 2A would overlap with the dotted traces (not shown). The scales are chosen so that the analytical WLC distributions are linearized (26). It is seen that the LSEC potential gives a strongly different distribution only for one base pair step, but for the DNA length corresponding to one helical turn the two distributions almost converge due to the central limiting theorem of the probability theory.

The above reasonings and Figure 2 illustrate two general conclusions. First, kinks should be common in strongly bent DNA, but they cannot affect the observable elastic parameters of free DNA because of their low statistical weight (36). Second, reversible temporary kinking due to a sigmoidal profile of *E*(*θ*) can qualitatively perturb the equilibrium statistics only for chain lengths comparable to that of the kink. Therefore, the base pair step kinkability cannot be responsible for perturbations at length scale beyond one helical turn. One cannot exclude, however, that kinks appear only on longer scales. For instance, the *E*(*θ*) profile can be flexed due to ion–DNA interactions across the B-DNA grooves, which requires more than one helical turn. Such kinking would not imply local melting or destacking and it can perturb the bend angle distributions on length scales of a few turns including the range from 3.5 to 10 nm where persistent non-Gaussian deviations were observed (47). Well-documented examples of ion-dependent DNA kinking on similar length scales exist in the literature (37,52).

### Statistics of DNA bending according to AFM

**Figure 3.**
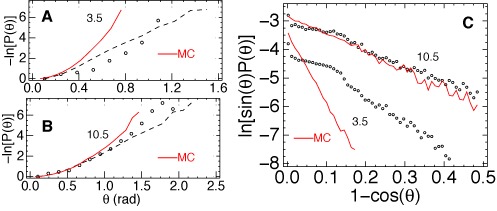
Probability distributions of DNA bending obtained with *l*_0_ = 3.5 nm. In all plots, experimental data obtained with the new tracing algorithm (open dots) are compared with the results of MC simulations of AFM experiments for the WLC model with *l*_*b*_ = 56 nm (solid lines). Panels (**A**) and (**B**) show negative logarithm of *P*(*θ*) for the angle *θ* between tangents separated by contour lengths 3.5 and 10.5 nm, respectively. Dashed traces display distributions evaluated with the original tracing algorithm (13). In panel (**C**) the small angle part of the same data is shown using coordinate scales that linearize analytical WLC distributions. The DNA lengths are shown near the plots in nanometers.

The defect of the earlier tracing procedure displayed in Figure 1A evidently degraded the accuracy of bend angles measured in short DNA. This particular DNA fragment, however, is just an example and it is difficult to estimate the overall impact of this feature upon the measured statistics. It turned out, that, with the divergence of DNA tracing reduced as in Figure 1C, deviations from the WLC model do not disappear. Two representative distributions of bend angles are analyzed in Figure 3. The AFM images of DNA were traced with the link length of *l*_0_ = 3.5 nm corresponding to one helical turn. Panel (A) reveals that for the minimal intersegment separation (3.5 nm) the new experimental plot significantly deviates from the reference WLC curve. The dashed line shows the distribution obtained with the original algorithm (13). It is nearly exponential in qualitative agreement with the LSEC hypothesis. With the new algorithm, the relative populations are increased for intermediate values of bend angles. As a result, the distribution is no longer exponential. Rather, its shape resembles the MC profile; therefore, the quantitative difference from the WLC model cannot be attributed to local kinkability. This feature is particularly clear in panel (C) where the distributions are compared using coordinate scales that linearize the small angle part of the corresponding analytical WLC curves. The experimental distribution, however, corresponds to a significantly lower *l*_*b*_ value. With increased DNA length, the AFM distributions rapidly converge to the MC plots. For the length of 10.5 nm (three helical turns) the experimental distribution agrees with the WLC model, with a negligible difference from MC simulations as regards the apparent *l*_*b*_ value.

**Figure 4.**
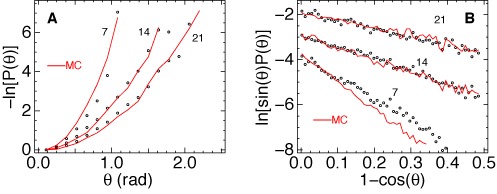
Probability distributions of DNA bending obtained with *l*_0_ = 7 nm. Panel (**A**) shows negative logarithm of *P*(*θ*) for the angle *θ* between tangents separated by different contour lengths. The dots show experimental data. The solid lines show results of MC evaluation of the same function for the WLC model with *l*_*b*_ = 56 nm. In panel (**B**) the small angle part of the same data is shown using coordinates that linearize WLC distributions. The corresponding DNA lengths are shown near the plots in nanometers.

With a two times larger link length (*l*_0_ = 7 nm) the improved algorithm shows no deviations from the WLC model. As seen in Figure 4, for the minimal intersegment separation (7 nm) the probability distribution agrees with the WLC prediction. The apparent *l*_*b*_ value is slightly lower than in MC simulations, but this cannot be considered as a deviation or an error because in both cases this value is somewhat larger than the long-chain limit. We can conclude, therefore, that already for two helical turns the double helical DNA in AFM experiments behaves in agreement with the WLC theory.

**Figure 5.**
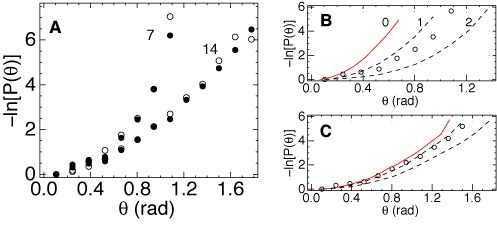
(**A**) Negative logarithm of *P*(*θ*) for 7- and 14-nm DNA evaluated from two independent sets of AFM images with pixels of 1.95 nm (open circles) and 3.8 nm (closed circles). The tracing link length *l*_0_ was 7 nm in both cases. (**B**,**C**) Experimental data from Figure 3A and B (open circles) are compared with MC simulations (dashed and solid lines) of AFM experiments with subsequent random shifting of bead positions as explained in the text. The corresponding shift amplitudes are displayed in panel (B) in nanometers.

Comparison of Figures 3 and 4 suggests that the very high apparent flexibility of 3.5 nm DNA is an experimental error. Otherwise we would have to assume that DNA is prone to correlated deformations such that strong bends in neighboring helical turns are compensated. Several possible sources of errors can be considered. First, the accuracy of the tracing algorithm may fall when the link length *l*_0_ becomes comparable to the DNA width in AFM images. This explanation was put forward earlier (47), however, the results shown in Figure 1 indicate that, with the refined tracing algorithm, the width of the bundle is determined by the tip rather than the width of the profile in Figure 1B. Second, the maximal width of the bundle is about one pixel, therefore, one can reasonably surmise that the accuracy of tracing is limited by the pixel density of the AFM image. To check this explanation the AFM experiments were repeated with two times lower pixel resolution using the same DNA and identical solvent conditions. The new set of images was digitized with *l*_0_ = 14 and 7 nm. If the accuracy is indeed limited by the pixel size, the new pixel of 3.9 nm, with *l*_0_ = 7 nm, should give a reduced accuracy similar to that for *l*_0_ = 3.5 nm in Figure 3. This prediction, however, appears completely wrong. A representative comparison is shown in Figure 5A. Using the same link length the experimental distributions from Figure 4A are almost exactly reproduced.

The last (and, probably, correct) explanation we considered attributes the loss of precision with reduced *l*_0_ to the DNA structure itself. The physical width of the double helix is 2 nm. This value is the diameter of an enveloping cylinder that passes through the phosphate groups of the canonical B-DNA. However, the axis of the enveloping cylinder and the centerline of an AFM image do not coincide. If we imagine that a straight B-DNA adsorbed on a surface is scanned using a hypothetical cantilever with the tip diameter of say 0.3 nm the height maxima would follow a wavy line undulating between the boundaries of the enveloping cylinder. Therefore, with any tip diameter, the axis of the double helix cannot be localized with the accuracy better than 2 nm. This feature introduces in the measured bend angles irremovable noise inversely proportional to the link length *l*_0_. To estimate the possible contribution of this noise in our data we randomized the DNA contours produced by MC simulations by shifting the computed bead positions along the bisector of the angle between the two adjacent links. The magnitude of the shift was evenly distributed on intervals ±0.5 and ±1 nm corresponding to amplitudes of 1 and 2 nm, respectively. Thus obtained new sets of DNA traces were processed with the same scripts as other data. The results shown in panels (B) and (C) of Figure 5 reveal that this rough modeling makes the MC traces very similar to the experimental data. Good quantitative agreement can be achieved for a shift amplitude between 1 and 2 nm.

## CONCLUSIONS

The experimental evidences of the apparently anomalous flexibility of short DNA can be partitioned in two qualitatively distinct sets. The first involves the data on high rates of DNA cyclization. It was always understood that these observations, regardless of experimental errors (36), could be rationalized without questioning the fundamental physics of the double helix by assuming a certain probability of strong bends due to melting bubbles, for instance (18,22). In contrast, to account for the bend angle distributions obtained by AFM (13,15), and the end-to-end distance distributions measured by small-angle X-ray scattering interference (53), one has to assume that complex long-range correlations of unclear physical origin occur along DNA. The results described here and in the previous report (47) prove that the AFM data should be excluded from the above list. Moreover, the accuracy of the method is sufficiently high to confirm that the WLC model remains valid at all DNA length scales where it can be compared with experimental data. Strictly speaking, for small length of about one helical turn, comparison of experimental DNA structures with the WLC model can never be accurate because non-planar conformations of neighboring base pairs, accompanied by regular rotation due to the helical twist, make the very definition of bending ambiguous unless the DNA fragment has an exact integral number of turns. Because of this same rotation the position of the centerline in the AFM images undulates, which makes accurate measurements of bend angles impossible. Our results prove that there is no detectable correlations in bending of neighboring DNA stretches for length scales beyond one helical turn. Accordingly, there is no chain length dependence of the apparent bending persistence length. If such correlations exist in free DNA they should be due to interactions that are incompatible with the specific conditions of the AFM experiments.
